# The effect of visual salience on memory-based choices

**DOI:** 10.1152/jn.00068.2013

**Published:** 2013-11-06

**Authors:** Arezoo Pooresmaeili, Dominik R. Bach, Raymond J. Dolan

**Affiliations:** ^1^Berlin School of Mind and Brain, Humboldt-Universität zu Berlin, Berlin, Germany;; ^2^Wellcome Trust Center for Neuroimaging, Institute of Neurology, University College London, London, United Kingdom; and; ^3^Department of Psychiatry, Psychotherapy, and Psychosomatics, University of Zurich, Zurich, Switzerland

**Keywords:** bottom-up attention, saliency, memory, choice

## Abstract

Deciding whether a stimulus is the “same” or “different” from a previous presented one involves integrating among the incoming sensory information, working memory, and perceptual decision making. Visual selective attention plays a crucial role in selecting the relevant information that informs a subsequent course of action. Previous studies have mainly investigated the role of visual attention during the encoding phase of working memory tasks. In this study, we investigate whether manipulation of bottom-up attention by changing stimulus visual salience impacts on later stages of memory-based decisions. In two experiments, we asked subjects to identify whether a stimulus had either the same or a different feature to that of a memorized sample. We manipulated visual salience of the test stimuli by varying a task-irrelevant feature contrast. Subjects chose a visually salient item more often when they looked for matching features and less often so when they looked for a nonmatch. This pattern of results indicates that salient items are more likely to be identified as a match. We interpret the findings in terms of capacity limitations at a comparison stage where a visually salient item is more likely to exhaust resources leading it to be prematurely parsed as a match.

decisions are often based on accessing representations from memory. Even the simplest form of memory-based decision, say deciding whether a bottle of wine is the same or different from a previously seen one at another store, involves a sequence of processing stages that entails specific form of information (Baddeley 2012). In its simplest form, this sequence entails encoding sensory information about the first stimulus (bottle of wine), its transformation into a stable working memory representation, recall of memory representation, e.g., tall dark bottles with a specific form, a comparison of the different items currently present in view that are each compared with the memory-coded item leading to a decision as to whether what we see and have “in mind” are similar or different. The multiplicity of processing stages and the limited processing capacity of our brain highlight the importance of selective attentional mechanisms in supporting optimal decision making.

Previous studies have suggested distinct but interrelated mechanisms through which attention can influence memory-based choices (Chun 2011; Cowan 2011). These studies mainly address capacity limitation of working memory and the ways in which allocation of visual attention impacts on this capacity (Luck and Vogel 1997; Zhang and Luck 2008). To this end, typically a multi-item stimulus display is used that is maintained in working memory across a variable delay. The classic finding is that a limited number of items (4) can be encoded and retrieved from memory (Cowan 2001), and successful retrieval of an item requires attentional orientation to that item during the encoding phase. Most of these studies propose that visuospatial attention is primarily involved during the encoding of information into the working memory (Schmidt et al. 2002; Vogel et al. 2006). Another view arising out of a number of recent elegant studies indicates that directing attention to a stimulus well after the encoding stage can affect the success with which an item is retrieved from working memory (Griffin and Nobre 2003; Kuo et al. 2012; Murray et al. 2013). Therefore, the effects of attention on memory-based choices seem also to extend to a maintenance and retrieval phase. Here, we ask whether it is possible that attention can have even a later impact, namely at a decision-making stage.

One obvious way to address this question is to manipulate the allocation of attention at the very stage when choices are made. If one uses this approach, it is crucial to ensure that the potential for visual attention to impact on earlier stages is controlled for or minimized such that only late effects are allowed to operate. In the present study, we adopted this approach and tested two specific mechanisms through which attention might influence memory-based choices. The first is related to findings showing that when items are compared with a memory template, a “matching” process takes place whereby a representation of the stimulus that matches the memory template is enhanced (Desimone 1996; Miller and Desimone 1994). Reorienting attention to a stimulus while this matching process is taking place may modulate the “priority” with which that stimulus enters this putative matching process or the “weight” that is assigned to it as being a match. Therefore, it is possible that attended items are more/less likely to be detected as a match to the memory due to prior/delayed entry or allocation of a higher/lower weight in a matching process. The final responses will depend on whether subjects are asked to find a “match/same” or a “nonmatch/different” item than the memorized template. A second possibility is that orienting visual attention to an item in the test array will override the memory-based comparison and bias an observer to select the attended item more/less often, irrespective of task requirements. This hypothesis holds that visual attention has a late “direct” influence on choices, presumably by giving more/less weight to the attended item during a final response-selection stage.

To test these alternatives, we designed a novel two-alternative forced-choice (2AFC) task (Fig. 1) wherein subjects decided which of two options had the same, or a different, orientation to that of a previously cued sample item. Visual attention was manipulated through varying the visual salience of the two test items such that they could be both similar to all of the other items in the display (no-salience, NS condition) on the one hand or alternatively either the matching or the nonmatching stimulus could be distinct from the rest of the items (match-salient, MS, and nonmatch-salient, NMS, respectively). Manipulation of the distinctness or salience of an object is an explicit and direct way to influence the allocation of attention since it is known that items that stand out in a scene attract bottom-up attention (Fecteau and Munoz 2006; Itti and Koch 2000; Treue 2003). Several aspects of the task were specifically tuned to minimize early perceptual and memory effects of attention. First, on each trial, only one item needed to be encoded into working memory, thereby avoiding a high memory load that promotes a need for attention at the encoding stage. Second, manipulation of visual attention followed item encoding into working memory and corresponded in time to when subjects compared the test array with the sample. Importantly, the visual salience of an item was uninformative in relation to a match or nonmatch. Third, time pressure to respond was minimal, and therefore speeded responses that depend on a fast accrual of information, known to be affected by attention, were not mandatory. Finally, the task was devised as a 2AFC task with suprathreshold stimuli that minimized demands on attention during sensory encoding of the test items.

**Fig. 1.**
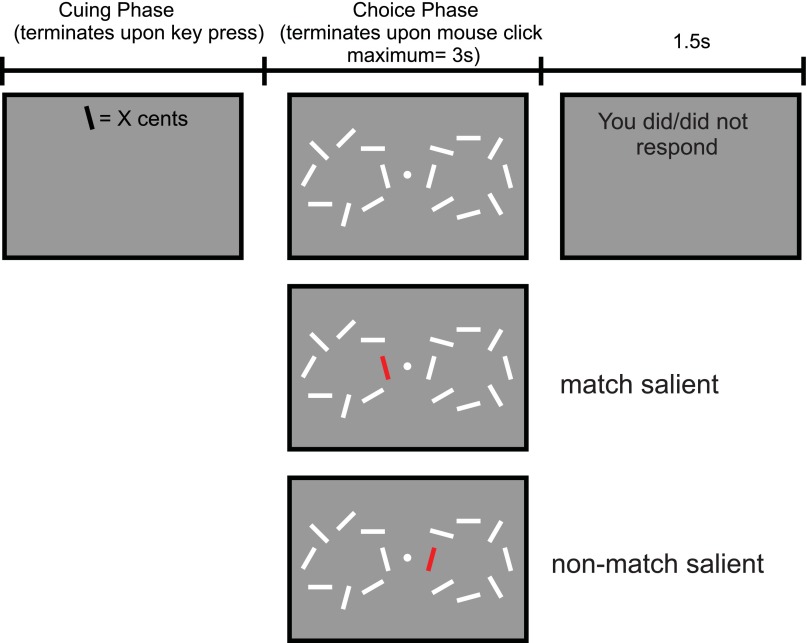
Behavioral task. *A*: at the beginning of each trial, a sample orientation and the amount of reward (low, 3¢, or high, 20¢) was displayed. Subjects pressed a button to proceed to the main task. The stimulus display contained a matching bar that had the same orientation as the sample bar and a nonmatching bar with a different orientation. These 2 bars were presented to the left and right of fixation point and were each embedded in an array of other randomly oriented bars. Only the bars that were on the horizontal meridian and closest to the fixation point can be the target, and all other bars were irrelevant but provided a context. Visual salience was manipulated by varying the color of the target and nontarget bar: trials in which both bars had the same color as the rest of the items are no-salience condition (NS), trials where either matching or nonmatching bars are salient are match-salient (MS) or nonmatch-salient (NMS), respectively. Subjects have 3 s to choose a stimulus either with the same (*experiment 1*) or different orientation (*experiment 2*) as the memorized sample via a mouse click.

We carried out two experiments where subjects either reported which item had the same or a different orientation than that of the sample. A direct impact of salience on choices would predict that in both cases subjects would choose an item more/less often when it is salient. Alternatively, if attention impacts the matching process, subjects are more/less likely to perceive the salient item as a match, and therefore their choices will be biased in opposite directions in these two tasks. Our results show that salience affects the matching process during a comparison stage such that salient items are more likely to be identified as a match to the sample.

## METHODS

### 

#### Participants.

We recruited 20 participants (9 women and 11 men, mean age = 26 yr) in our 1st experiment and 18 participants (12 women and 6 men, mean age = 25 yr) in our 2nd experiment. Subjects gave informed oral and written consent for their participation. The study was approved by the local ethics committee of the Charité - University Medicine Berlin.

#### Behavioral paradigm.

At the beginning of each trial, subjects first saw the sample orientation and the amount of reward available (Fig. 1). The trial then commenced with subjects having to decide which of the two bars (match and nonmatch), located to the left and right of a fixation point, was the same (*experiment 1*) or different (*experiment 2*) to a sample orientation. Since the effect of visual salience is strongest in crowded displays (Treisman and Gelade 1980), we embedded the matching and nonmatching bars within an array of oriented bars to enforce an effect of salience. However, the task of subjects did not differ from that of a typical 2AFC task since the possible target locations were restricted to the two positions on the horizontal meridian closest to the fixation point. Consequently, bars positioned at other locations were task-irrelevant and only provided a spatial context within which the target bar could stand out.

We manipulated salience by varying the color of the target and nontarget bars across trials. Trials in which both bars had the same color as the rest of the items (white) constituted NS trials, whereas trials in which either the matching or the nonmatching bar had a different color (red) constituted MS or NMS trials, respectively. The order of presentation was fully randomized, and all conditions were counterbalanced.

The stimulus display was in view for maximum 3 s. Subjects indicated their response by moving the mouse cursor and clicking on a bar that was the same (*experiment 1*) or different (*experiment 2*) from the sample orientation. A trial ended as soon as one of the bars was selected, and thereafter it was indicated whether subjects had responded, but no feedback regarding the accuracy or speed of the choices was provided.

We varied the reward level that subjects could obtain on each trial (either 3 or 20¢). Reward levels were varied randomly and were unrelated to task difficulty and counterbalanced across conditions. Across all of the pilot and final experiments, we failed to observe a consistent effect of reward. Therefore, for all subsequent analyses, we pool data across the 2 reward levels to increase our analysis power. For each subject, a total number of 600 trials were collected, corresponding to 40 trials per stimulus condition (5 orientations and 3 salience levels).

#### Stimuli and apparatus.

Stimuli were produced with MATLAB and the Psychophysics Toolbox (Brainard 1997; Pelli 1997). Stimuli were displayed on a 21-in. calibrated CRT monitor (Barco) with 1,152- × 864-pixel resolution and a refresh rate of 85 Hz and were viewed at a distance of 57 cm. The stimulus display (gray background; luminance = 30.2 cd/m^2^) contained 2 arrays of tilted bars (8 bars; width = 1 visual degree, height = 4.5 visual degrees, luminance contrast = 63%) positioned around 2 circular areas on the left and right side of a fixation point. Each circular array had a radius of 5°, and its center was positioned at 9° eccentricity from the fixation point. The match and nonmatch bars were positioned on the horizontal meridian at 4° eccentricity; therefore, they were the closest points to the fixation point on each array. In NS trials, all of the bars were white [red, green, and blue values (RGB) = 255, 255, 255], whereas in MS and NMS trials, one of the bars was red (RGB = 255, 0, and 0). The sample bar displayed at the beginning of each trial was always black (RGB = 0, 0, and 0) and was otherwise identical to the stimulus bars. The orientation of sample bars was randomly varied between 15 and 180°. Task difficulty was manipulated by randomly varying the orientation difference between the matching and nonmatching bars (Δθ°) between 1 and 30° (1, 5, 10, 15, and 30°).

#### Analysis of the performance data.

We analyzed the behavioral data both in terms of probability of choice (irrespective of whether they were correct) and probability of correct responses. Probability of choice was computed based on the proportion of trials in which one of the two test stimuli was selected. To measure the choice bias toward or away from a stimulus as a function of orientation match (*experiment 1*) or mismatch (*experiment 2*) with the sample, a sigmoid function in the following form was fitted to the data:

$$P(\Delta \theta )=\frac{1}{\exp\left[\frac{-\left(\Delta \theta -a\right)}{b}\right]}$$

where *P* denotes the probability of choice, Δθ is the orientation difference between a stimulus and the memorized sample, *a* is the horizontal offset, and *b* is the steepness of the sigmoid. The fitting was implemented in MATLAB using a maximum likelihood method.

## RESULTS

**Fig. 2.**
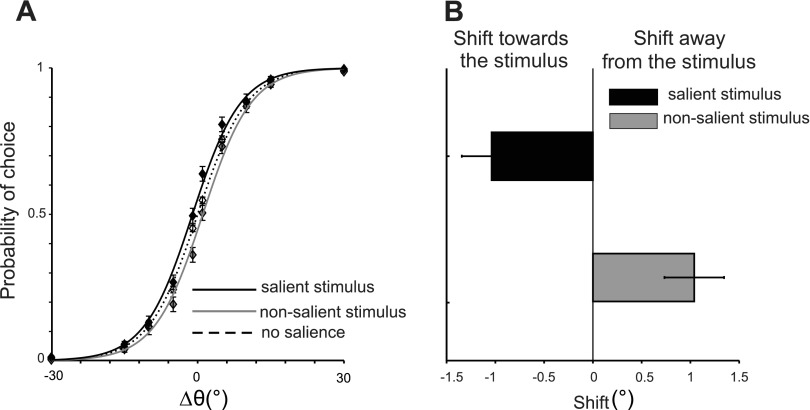
Effect of salience on choice when subjects search for a match (*experiment 1*). *A*: probability of choosing a stimulus is shown as a function of orientation difference (Δθ°) from the sample orientation. The sign of Δθ° indicates the match with the sample with Δθ° < 0 indicating that a stimulus did not match the sample orientation and Δθ° > 0 indicating a match in orientation. Probability of choice varied as a function of orientation similarity between the 2 bars and their match to the sample. The dashed curve corresponds to psychometric functions in NS condition. Solid curves correspond to when a stimulus is salient (black) or NS (gray). Stimulus salience produces a choice bias as demonstrated by a horizontal shift of the psychometric curves. *B*: choices are shifted toward the salient stimulus (leftward shift, negative offset) and away from the NS stimulus (rightward shift, positive offset).

In *experiment 1*, subjects were asked to find the test bar that matched the orientation of the sample. Figure 2 illustrates how the probability of choosing a stimulus varied as a function of its orientation difference from the sample bar (Δθ°). Note that since one of the test bars was a match and the other a nonmatch, the absolute value of Δθ° also indicates the degree of similarity between the two test stimuli. The sign of Δθ° indicates whether a stimulus was the same (Δθ° > 0) or different (Δθ° < 0) from the memory template.

In the absence of salience, probability of choice increased with Δθ° and was symmetrical around 0 (mean horizontal offset of the sigmoid = −0.0003). When a stimulus was salient, the subject chose it more often as if it had been a match to the sample orientation. This choice bias is demonstrated by a leftward shift of the psychometric function (black curve in Fig. 2*A*, mean horizontal offset of the sigmoid = −1.04). Conversely, a NS stimulus was selected less often, resulting in a rightward shift of the psychometric function. Note that the shift of psychometric curve away or toward the salient stimulus is equal in two directions because subjects either chose one or the other stimulus. This shift was significantly different from the NS condition (*P* = 0.003, paired *t*-test). The steepness of the psychometric function was not significantly different between the salient and NS conditions (mean = 4.85 and 5, respectively; *P* = 0.67, paired *t*-test). These results indicate that subjects had a tendency to select the salient item more often, in effect acting as if it had matched the sample orientation.

**Fig. 3.**
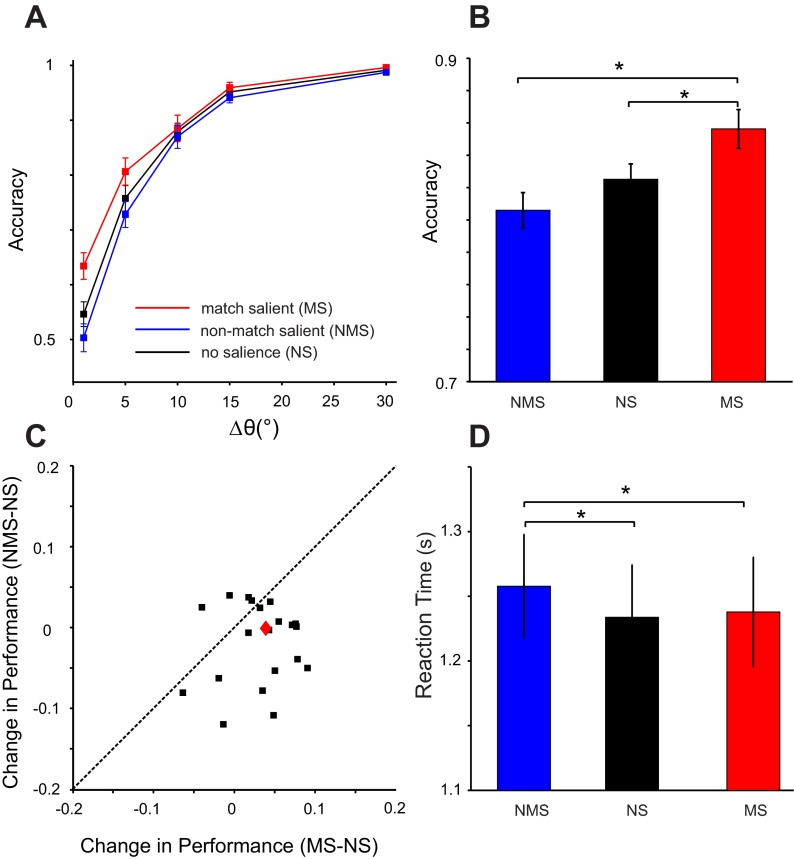
Accuracy and reaction times (*experiment 1*). *A*: probability of correct responses (accuracy) as a function of Δθ° between the 2 test bars for NS (black), MS (red), and NMS (blue) conditions. *B*: average accuracy across all orientation differences. *C*: change in accuracy in MS and NMS conditions compared with NS trials for all of the subjects. The black squares are the data of individual subjects, and the diamond is the average across all subjects. *D*: average reaction time across all orientation differences. Error bars are standard error of the mean; asterisks denote significance (*P* < 0.05) as measured by pairwise *t*-tests.

Figure 3*A* shows the performance of subjects quantified as the probability of selecting a stimulus when it had the same orientation as the sample. Averaged across all Δθ° levels, subjects were overall better (mean accuracy = 0.85) when the matching item was salient and performed worse when a nonmatch was salient (mean accuracy = 0.8) compared with the NS condition (mean accuracy = 0.82; *P* = 0.003 and 0.1, for the comparison of MS vs. NS and NMS vs. NS, respectively). Analysis of the reaction times across all Δθ° showed a significant increase of reaction times in NMS compared with NS condition, whereas reaction times were not different in MS and NS (average reaction times: 1.26, 1.24, 1.23 s in NMS, NS, and MS; *P* = 0.02 and 0.63 for the comparison of NMS vs. NS and MS vs. NS, respectively). These results suggest that when subjects are asked to find a matching item, they exhibit a tendency to select a visually salient item as a match. This tendency translates to a gain in accuracy when the salient item is indeed a match but results in a cost, especially in terms of reaction times, when the salient item is a nonmatch.

These findings support the hypothesis that visual salience can influence memory-based choices. However, it is not obvious whether this effect is exerted at the final choice level or at an earlier comparison stage, when test stimuli are compared with the memory template. To differentiate between these mechanisms, we carried out a second experiment, where we asked subjects to identify the item that had a different orientation than that of the sample stimulus.

**Fig. 4.**
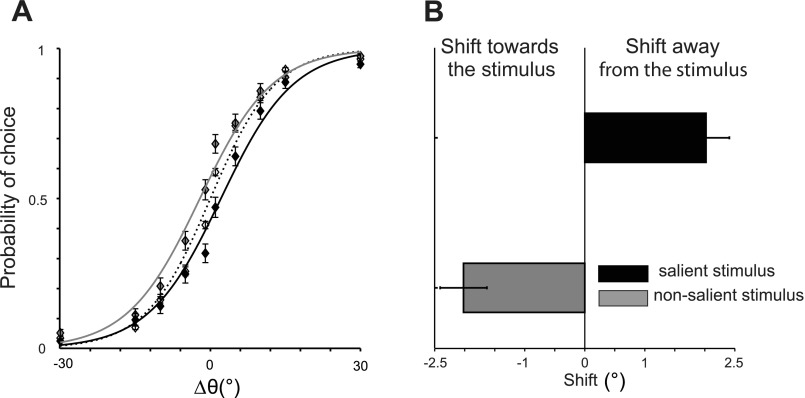
Effect of salience on choice when subjects search for a nonmatch (*experiment 2*). *A*: probability of choosing a stimulus as a function of Δθ° from the sample orientation. The sign of Δθ° indicates the match with the sample, with Δθ° < 0 indicating that a stimulus matched the sample orientation and Δθ° > 0 indicating that it had a different orientation. Probability of choice varies as a function of orientation similarity between the 2 bars and their match to the sample with higher choice probabilities for more dissimilar items. The dashed curve corresponds to psychometric functions in NS condition. Solid curves correspond to when a stimulus is salient (black) or NS (gray). Stimulus salience produces a choice bias as demonstrated by a horizontal shift of the psychometric curves. *B*: choices are shifted toward the NS stimulus (leftward shift, negative offset) and away from the salient stimulus (rightward shift, positive offset).

**Fig. 5.**
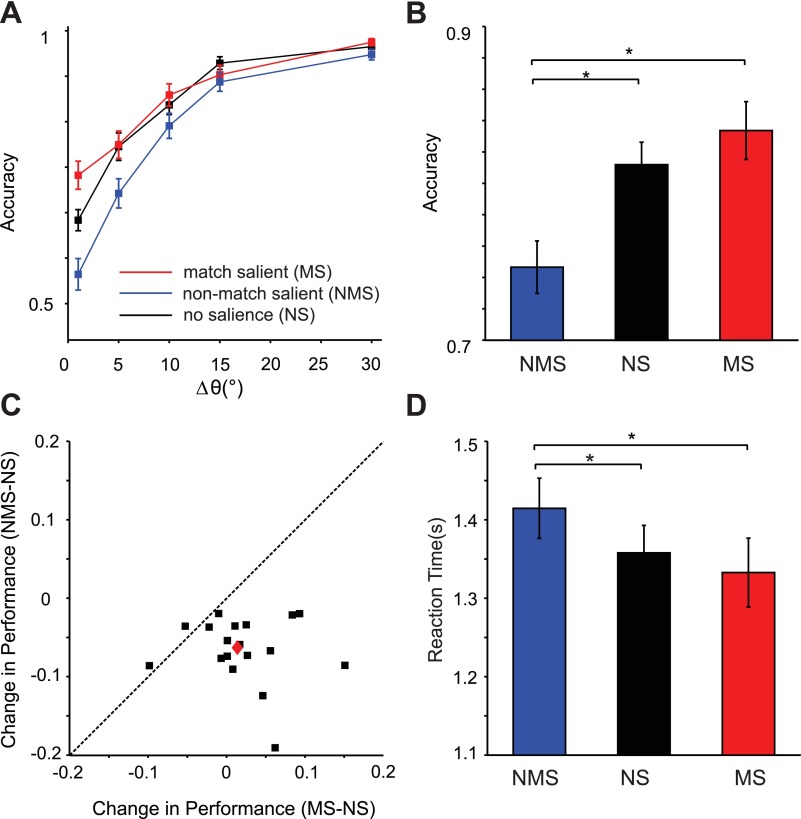
Accuracy and reaction times (*experiment 2*). *A*: probability of correct responses (accuracy) as a function of Δθ° between the 2 test bars for NS (black), MS (red), and NMS (blue) conditions. *B*: average accuracy across all orientation differences. *C*: change in accuracy in MS and NMS conditions compared with NS trials for all of the subjects. The black squares are the data of individual subjects, and the diamond is the average across all subjects. *D*: average reaction time across all orientation differences. Error bars are standard error of the mean; asterisks denote significance as measured by pairwise *t*-tests.

Figure 4 illustrates the probability of choices in the second experiment. In this figure, positive Δθ° indicates that a stimulus was a nonmatch, and negative Δθ° corresponds to when it was a match to the sample orientation. Subjects selected a stimulus less often when it was salient, as indicated by a rightward shift of the psychometric curve (black curves), whereas a NS item was more likely to be selected as shown by a leftward shift of the curve (gray curve). The horizontal shift of the curves was larger than that of the first experiment, albeit in an opposite direction, and was significantly different from the NS condition (mean horizontal shift compared with NS condition = 2.02; *P* < 10^−4^, paired *t*-test). The differences in steepness of the psychometric curves were not significant (average steepness: 6.5 and 7.2 in NS and stimulus-salient conditions, respectively; *P* > 0.05, paired *t*-test). These results indicate that subjects were less likely to select a salient item when they had to find a test stimulus with a different orientation. It follows that subjects should miss some trials where the stimulus is salient and is indeed different from the sample. As demonstrated in Fig. 5, this is in fact what we found: subjects' performance both in terms of accuracy and reaction times deteriorated when a nonmatching stimulus was salient (average accuracy = 0.74 and 0.81 in NMS and NS condition, respectively, *P* < 10^−5^, and average reaction time = 1.41 and 1.35 s, *P* = 0.003, paired *t*-test). There was a small trend toward better performance in MS condition compared with NS condition, but the differences were not significant (average accuracy = 0.83 and 0.81 in MS and NS condition, and average reaction time = 1.33 and 1.35 s, respectively, both *P* > 0.05, paired *t*-test). Comparison of the two experiments also reveals that finding a nonmatch is generally more difficult than finding a match, as demonstrated by lower accuracies and higher reaction times in *experiment 2*. The results of the two experiments taken together do not support the notion that visual salience affects the final stage of the task where one of the two stimuli is selected. Rather, it seems that salience renders a stimulus more likely to be identified as a match to that of a memory template.

## DISCUSSION

The present study demonstrates that visual bottom-up attention, as in visual salience, influences human memory-based choices even when item salience is task-irrelevant. This effect is evident in a tendency of subjects to identify a salient stimulus as a match, therefore selecting it more often in a match-to-sample task and less often so in a nonmatch-to-sample task.

In our experiments, we controlled or minimized attentional demands at early encoding and retrieval phases so that later effects on a comparison stage or a final selection stage could be isolated. Attentional biases in choice have been reported in a number of recent studies (Krajbich and Rangel 2011; Krajbich et al. 2010, 2012; Lim et al. 2011; Mormann et al. 2012; Wittmann et al. 2008) where the pattern of visual fixations (Krajbich et al. 2010) and visual salience (Mormann et al. 2012) affected subjects' value-based decisions. In these studies, a selection bias was observed wherein subjects chose items that had been fixated for longer or were more salient but less preferred. This emerging view that attention can guide decisions motivated us to test the possibility that visual salience produces a selection bias in memory-based decisions such that salient items are selected more often even when they are not relevant. In line with these findings, we also found evidence for an impact of visual salience on choices in both of our experiments, thus extending the previous findings to memory-based decisions. The two tasks that we used both involved a matching stage where subjects try to find an item that matched the memory template. This was also the case in the second experiment, where subjects were required to select a nonmatch. Instead of looking for a nonmatch, subjects first tried to find the matching item and then select the other item (as reported by all of the subjects when debriefed about their strategies). As such, the two tasks were identical up to the final selection phase when either the match (*experiment 1*) or the nonmatch (*experiment 2*) was selected. Since the bias in final choice was in opposite directions in these two experiments, the possibility that salience-related choice biases occur at the final response selection stages is strongly ruled out. We therefore propose that, at least in the context of our experiments, attentional choice biases in memory-based decision making operate before final selection, perhaps through invoking a matching bias, whereby attended or more salient items are more often matched to a memory template.

How exactly such a putative matching process might operate? According to the biased competition theory (Desimone and Duncan 1995; Desimone 1998), working memory representations act as top-down control mechanisms that bias selection to matching stimuli in the visual field. It is often assumed that matching is an automatic process that occurs in a parallel, preattentive fashion and even occurs when matching is completely task-irrelevant. Alternatively, early item-recognition studies (Sternberg 1966) had proposed that matching items to memory is a serial comparison process with an exhaustive memory scan coupled with a self-terminating visual scan. In this scheme, when a match is found, search terminates, and no further visual item is processed. The idea of serial matching to memory is supported by studies showing that visual inputs can be matched to only one memory template at a time, which suggests that matching occurs in a serial and capacity-limited manner (Houtkamp and Roelfsema 2009). Our data are in line with this theoretical framework and in fact demonstrate an extreme case of capacity limitation during matching process. Orienting attention to a test item accelerates the sensory processing of that stimulus and thereby grants prioritized entry of the attended stimulus into the matching process. If matching process has a capacity limitation, it could be prematurely “filled” with the attended item. As a result, a more exhaustive match to all of the other items in the display is ceased, and the attended item is parsed as being a match. This proposal can explain the pattern of our results: a serial matching process that scans the scene until a match is found can explain why reaction times are shorter when the matching item is salient (compared with NMS condition), whereas the premature termination of matching can explain choice biases and accuracy data.

The effect of visual attention on memory-based tasks has been mainly investigated by manipulation of top-down attention. Three recent studies have applied manipulation of bottom-up visual salience. In two of these studies (Fine and Minnery 2009; Santangelo and Macaluso 2013), visual salience of memory items has been varied, and it has been found that visually salient items are better retrieved from memory. Interestingly, Melcher and Piazza (2011) showed that as the salience of an item encoded in the working memory increases, the memory performance for all other less-salient items decreased, suggesting that visual salience of items influences capacity limits of visual working memory. Our results are in line with this suggestion and point to a similar mechanism that may shape capacity limits during comparison stage of working memory tasks. However, these findings could be integrated within a general framework of a master salience map that integrates top-down and bottom-up information and impacts on choices at various stages.

Another line of related research pertains to the effect of so-called retro-cues (Griffin and Nobre 2003). Retro-cues appear well after the memory array and direct the visual attention to one item in the array. It has been shown that retro-cued and therefore attended items are remembered better compared with the unattended items. Although there is a similarity in our findings to retro-cue studies, there are also a number of key differences. First, a retro-cue is usually only effective if it appears at the delay interval between the memory and test array. In fact, a cue at the time of test array (called postcue) has no effect on performance (Sligte et al. 2008). Second, retro-cues usually direct attention to a relevant feature of the memory template, most often its spatial location (Griffin and Nobre 2003; Kuo et al. 2012), as well as to other features (e.g., color; Pertzov et al. 2013). Our manipulation of salience, however, was completely orthogonal to the task and fully uninformative about the “to be reported” feature (orientation in our experiments). However, it could still be argued that we orientated attention to one feature of an object and thereby granted privileged “access” to all of its memorized features, as demonstrated by higher accuracy and shorter reaction times for MS compared with the NMS condition. We cannot rule out this possibility, although we take a view that our effects are better explained by a choice bias rather than a change in sensitivity of memory retrieval. This distinction could be better addressed in future studies with a design that also includes zero differences (Δθ° = 0) between memory template and test array and measures all response options in a signal-detection framework.

What are the neuronal mechanisms that underlie the effect of salience on memory-based choice? Previous studies have shown that neurons in frontal and parietal cortex contain a salience map where bottom-up salience and task-relevant information of different items in visual space are represented (Gottlieb et al. 1998; Katsuki and Constantinidis 2012; Thompson and Bichot 2005). On the other hand, electrophysiological studies have shown that comparison of visual inputs with memory templates involves the same cortical areas that underlie visual perception (Hayden and Gallant 2013; Hussar and Pasternak 2013; Lui and Pasternak 2011; Miller et al. 1991; Miller and Desimone 1994). Although we cannot point to a specific neuronal mechanism where salience and matching signals are integrated, we can speculate that such an integration needs salience signals to be delivered to comparison areas and that matching signals are weighted based on the salience information. These processes may be implemented via back-propagation of salience signals to sensory areas or by downstream areas that have access to both signals and implement the comparison process (e.g., in dlPFC, as shown recently; Suzuki and Gottlieb 2013). Recently, computational models have integrated different stages of working-memory decisions and have explained them in terms of elementary neuronal computations that can occur in a single cortical area, thus removing the need for a hierarchical organization of memory-based choices (Engel and Wang 2011). These models together with future behavioral investigations of the effects of attention on memory-based choices will be instrumental for understanding “same/different” decisions.

## GRANTS

This work was performed while R. J. Dolan was a Visiting Einstein Fellow at the Humboldt-Universität, Berlin School of Mind and Brain and was supported by Wellcome Trust Senior Investigator Award 098362/Z/12/Z to R. J. Dolan.

## DISCLOSURES

No conflicts of interest, financial or otherwise, are declared by the author(s).

## AUTHOR CONTRIBUTIONS

A.P., D.R.B., and R.J.D. conception and design of research; A.P. performed experiments; A.P. analyzed data; A.P., D.R.B., and R.J.D. interpreted results of experiments; A.P. prepared figures; A.P. drafted manuscript; A.P., D.R.B., and R.J.D. edited and revised manuscript; R.J.D. approved final version of manuscript.
